# An overview of electronic cigarette use and consequences among adolescents.

**DOI:** 10.36416/1806-3756/e20250137

**Published:** 2025-05-21

**Authors:** Miguel Ângelo Uflacker Lutz de Castro, Laura Menestrino Prestes, Gabriela de Azevedo Bastian de Souza, Maria Paula de Carli Hanel, Leonardo Araújo Pinto, Débora Carla Chong e Silva

**Affiliations:** 1. Grupo de Pesquisa em Epidemiologia e Genética das Doenças Respiratórias da Infância, Pontifícia Universidade Católica do Rio Grande do Sul.; 2. Programa de Pós-Graduação em Medicina - Pediatria, Escola de Medicina, Pontifícia Universidade Católica do Rio Grande do Sul.; 3. Departamento de Pediatria, Pontifícia Universidade Católica do Paraná.; 4. Programa de Pós-Graduação em Saúde da Criança e do Adolescent, Universidade Federal do Paraná.

## INTRODUCTION

Electronic nicotine delivery systems (ENDS), commonly referred to as electronic cigarettes or “e-cigarettes,” function by delivering aerosolized particles containing nicotine or other substances to the user.^1^ First introduced to the U.S. market around 2007, these devices emerged as an alternative for individuals attempting to quit traditional cigarettes.^2^ However, they have been shown to be equally harmful-if not more so-as they deliver higher levels of nicotine along with other potentially toxic chemicals, such as dichloromethane, acetaldehyde, acrolein, nitrosamines, and various agents that remain insufficiently characterized in the scientific literature.^3^ Consequently, another significant concern is the limited research on these devices and their long-term effects, particularly given their relatively recent introduction to the Brazilian market.^2^ Therefore, understanding the factors contributing to their increased use among adolescents, the short- and long-term effects of such use, and the broader context of the issue in Brazil warrants further investigation. 

## ARE ENDS BETTER THAN CONVENTIONAL CIGARETTES?

E-cigarettes are often marketed as a safer alternative to traditional cigarettes. Unlike conventional tobacco products, ENDS operate without combustion, thereby reducing the release of numerous toxic substances associated with smoking-related health issues.^1^ For instance, emissions from e-cigarettes typically contain lower levels of particulates and carcinogenic compounds compared to traditional cigarette smoke. However, the perception that ENDS are significantly safer remains a topic of ongoing debate. As relatively recent innovations, their long-term health effects are not yet fully understood, resulting in an incomplete and evolving body of evidence.^2^ Moreover, inconsistencies in product labeling and wide variability in nicotine and chemical content across different brands and models further complicate efforts to assess their safety. While ENDS may produce fewer harmful emissions than traditional cigarettes, they still expose users to nicotine-a highly addictive substance-and other potentially hazardous chemicals, raising concerns about their overall impact on health.^1^

## WHY ARE ENDS DANGEROUS?

ENDS are associated with several significant health risks. One of the primary concerns is their efficient delivery of nicotine, which can lead to rapid dependence and adverse health effects, such as increased heart rate and blood pressure. In extreme cases, nicotine toxicity may result in seizures, respiratory failure, or even death. The vapor produced by ENDS contains various harmful chemicals, including ultrafine particles, volatile organic compounds, and carbonyl compounds-some of which have been detected at levels comparable to those found in conventional tobacco smoke.^1^ These substances can irritate the respiratory tract, induce oxidative stress, and trigger inflammation. Moreover, flavoring agents commonly used in ENDS liquids, such as cinnamon, have demonstrated cytotoxic effects, raising concerns about the long-term consequences of inhaling such additives.^3^ Although ENDS emit fewer harmful substances than traditional cigarettes, secondhand vapor still exposes non-users to potentially dangerous chemicals, the full extent of which remains unclear. Furthermore, the appealing packaging and flavors of ENDS liquids present a serious hazard, particularly to children, as accidental ingestion can lead to severe health complications.^1^

## ADOLESCENTS AS MAIN USERS OF ENDS

With the rise in e-cigarette use, one population of particular concern is adolescents. A recent systematic review involving individuals aged 8 to 20 years from 69 countries and territories reported a pooled prevalence of 17.2% for ever-use (any lifetime use) of ENDS and 7.8% for current use (use within the past 30 days).^4^ ENDS are as addictive as conventional cigarettes and can cause neurocognitive effects, including impaired reflexes, deficits in attention and reasoning, and mood disorders. Furthermore, nicotine exposure at a young age can result in permanent brain damage, increase the risk of addiction, and contribute to sustained tobacco use into adulthood.

## CONSEQUENCES OF ENDS USE

ENDS pose significant health risks due to their chemical composition and the interactions these substances have within the human body. For example, e-cigarettes contain propylene glycol and vegetable glycerin-compounds that are highly toxic to lung cells. Generally, the greater the number of ingredients, the higher the potential harm to health.^1^ The consequences of ENDS use can be broadly categorized into five main areas: respiratory problems, cardiovascular damage, nicotine addiction, mental health issues, and substance abuse. 

[Table t1]

**Table 1 t1:** Health risks and management strategies related to e-cigarette use.

CONSEQUENCE	DESCRIPTION	HOW TO MANAGE
RESPIRATORY PROBLEMS	On usage, one inhales an aerosol laden with chemicals that can be harmful to the lungs. These agents travel deep within the lungs, causing irritation and inflammation. With time, this may lead to breathing disorders, including cough, wheezing, and asthma.	Discontinuing the use of all vaping products to prevent more damage is the main goal in management. Besides that, doctors are able to treat acute respiratory symptoms through prescribing medications such as bronchodilators, corticosteroids, or antibiotics. If breathing is severely compromised, the doctor may recommend hospitalization and oxygen therapy.
CARDIOVASCULAR PROBLEMS	Nicotine has a deleterious effect on cardiovascular health: it makes the heart beat faster and raises blood pressure. Long-term use of e-cigarettes with nicotine may be a risk factor for cardiovascular disease, which results from plaque build-up in arteries.	Other than stop vaping, treating acute cardiovascular symptoms associated with ‘vaping’ using beta-blockers, anti platelet agents, statines and ACE inhibitors is one of the most important ways to manage. Also, adopting a healthier lifestyle with regular exercise and stress reduction.
NICOTINE ADDICTION	It is able to quickly produce a great feeling of pleasure in the brain, thus carrying with it the sense of reward and reinforcement. The potential for strong nicotine dependence makes quitting electronic cigarettes very difficult for many individuals.	Treatment for mental health issues, nicotine addiction and vaping-related substance abuse requires an integrated approach starting with banning the use of any vape with nicotine replacement therapy, approved cessation programs and supporting therapy.
MENTAL HEALTH ISSUES	When it comes to emotional well-being, teenagers who vape may have a much more challenging time fending off anxiety and depression. Heads up, nicotine from e-cigarettes also plays a role in regulating neurotransmitters in the brain that control mood and increase the risk of anxiety and mood disorders.	In this context, emotional problems related to e-cigarettes need to be approached through therapies, like Cognitive behavioral therapy (CBT), relaxation techniques or if needed medication prescribed by a medical expert, such as psychiatrists.
RISK OF SUBSTANCE ABUSE	Research findings show that adolescents who engage in e-cigarette usage are more likely to use other tobacco products and illicit drugs. This increased likelihood of substance use is often attributable to the social and environmental factors that usually the use of e-cigarettes, such as exposure to peers who smoke other illicit substances and a tendentious propensity for risk-taking.	Expert counselling, support groups and for the more serious cases rehabilitation programmes can reduce risk for substance abuse. Healthy lifestyle, balanced diet, quality sleep and exercise also play a role in overall wellness and recovery. Consult with emergency help in case of acute mental health symptoms.

## WHAT IS THE CONTEXT OF ENDS IN BRAZIL?

The sale, importation, storage, transportation, and advertisement of e-cigarettes in Brazil have been prohibited by the National Health Surveillance Agency (ANVISA) since Collegiate Board Resolution (RDC) No. 46 of 2009. In 2024, the regulation was reviewed and reaffirmed by the Brazilian government with even stricter measures, including a ban on the manufacture and distribution of ENDS within the national territory.^5^ It is also important to note that electronic cigarettes are subject to Decree-Law No. 8.262 of 2014, which prohibits the use of any smoking device in enclosed public or private collective spaces.^6^

In 2022, the Covitel study was conducted in Brazil to estimate the prevalence of e-cigarette use among Brazilian adults. The study found that among 1,800 individuals, the prevalence of current commercial cigarette smoking was 12.2%. Furthermore, young adults aged 18 to 24 had the highest rates of e-cigarette experimentation, with usage being more common in the Central-West region and among individuals with higher levels of education.^6^

## CONCLUSION

In summary, while often considered a healthier alternative to traditional cigarettes, ENDS are, in fact, equally harmful-or potentially more so. In addition to causing nicotine dependence, they are associated with respiratory issues, cardiovascular problems, and negative impacts on mental health. Although the sale of these devices is prohibited in Brazil by ANVISA, their growing popularity among adolescents underscores the urgent need for both educational initiatives and strengthened regulatory measures.
